# Can “In–Out–In” posterosuperior screws meet nutrient foramina in patients with femoral neck fractures?

**DOI:** 10.1186/s13018-023-03936-w

**Published:** 2023-07-03

**Authors:** Shenghui Wu, Shitong Zhao, Aikebaier Aisikaer, Xiaozhong Zhu, Yu Miao, Guangyi Li, Yingqi Zhang, Jiong Mei

**Affiliations:** 1 Department of Orthopedic Surgery, Shanghai Sixth People’s Hospital Affiliated to Shanghai Jiao Tong University School of Medicine, Shanghai, China; 2 Department of Orthopedic Surgery, Tongji Hospital, Tongji University School of Medicine, Shanghai, China

**Keywords:** Femoral neck fracture, Posterosuperior screw, Fluoroscopy, Nutrient foramina, Damage risk assessment

## Abstract

**Background:**

The “In–Out–In” (IOI) posterosuperior screw was common in screw fixations of femoral neck fractures. The impacts of the IOI screw on the blood supply of the femoral head have not yet been clarified. The nutrient foramen was damaged when the screw was present in their corresponding cortex surface. This study aimed to evaluate the damage degrees of the nutrient foramina in the femoral neck as the IOI posterosuperior screw was placed in different posterosuperior locations.

**Methods:**

One hundred and eight unpaired dry human cadaveric proximal femurs were scanned by a three-dimensional scanner. Digital data obtained from the proximal femur surface were employed for subsequent analysis. All nutrient foramina in the femoral neck were identified and marked in each subject. A simulation of the anteroposterior, lateral, and axial views was then performed, and regions of interest (ROIs) for IOI posterosuperior screws, with 6.5 mm diameter, were determined in the posterosuperior femoral neck on the axial graphs. Nutrient foramina were counted and analyzed in ROIs and femoral neck, and its damage from the IOI posterosuperior screw was also calculated in different conditions of screw placement. Paired *t*-tests were used for comparative analyses before and after damage.

**Results:**

Most nutrient foramina were located in the subcapital region and the least in the basicervical region in the femoral neck, while the most were located in the transcervical and the least in the subcapital in the ROIs. In addition, most nutrient foramina in ROIs were located in the superior–posterior area of the femoral neck. There were four main locations of IOI posterosuperior screws where the decrease in the nutrient foramina was statistically significant (*P* < 0.01). The risk zone determined by these locations was located in a posterosuperior square of ROIs with an edge length of 9.75 mm.

**Conclusion:**

To minimize iatrogenic damage to the blood supply of the femoral head, screw positions could be assessed in anteroposterior and lateral radiographs using a risk zone. The IOI posterosuperior screw in ROIs can be applied to fix femoral neck fractures when feasible in clinical practice. This study could provide surgeons with more alternatives for screw placement in the posterosuperior femoral neck.

## Background

Femoral neck fractures are common hip trauma, and their incidence increases with population aging and traffic accidents. Multiple cannulated screws may be used to fix femoral neck fractures. The “In–Out–In” posterosuperior screw may penetrate the cortex anatomically, but not fluoroscopically, because of the specific morphology of the femoral neck, with a reported incidence of 70% [1, 2]. There were several novel fluoroscopic views and methods for the safe placement of the posterosuperior screw in femoral neck fracture fixation, but the “In–Out–In” screws in the posterosuperior femoral neck may not absolutely be avoided [3–8]. In addition, additional operations based on the above innovations may require increased procedure time and fluoroscopy time, compared with traditional fluoroscopy. Therefore, it is necessary to assess the effects of the “In–Out–In” posterosuperior screw on femoral neck fractures according to the traditional anteroposterior and lateral radiographs by C-arm machines.

An ideal design of fracture fixation can provide sufficient biomechanics, with no interruption of blood supply. A superior biomechanical property of fixation of femoral neck fractures can be attributed to the “In–Out–In” posterosuperior screw in inverted triangular screws [9]. To date, it was still unclear whether the “In–Out–In” posterosuperior screw disrupted the blood supply to the femoral head. When the “In–Out–In” posterosuperior screw was located in an extraosseous position, it was controversial whether the extraosseous vessels were damaged. However, the nutrient foramina in the corresponding cortex surface in the femoral neck were damaged due to the presence of the screw. In the femoral neck, the subcapital region was the most distributed site of the nutrient foramina, most of which were located in the posterosuperior area [10]. Thus, identifying the damage degrees of the nutrient foramina in different placements of the “In–Out–In” posterosuperior screw in the femoral neck is urgently needed to intraoperatively protect the blood supply of the femoral head, thus reducing the risk of femoral head osteonecrosis.

This study aimed to evaluate the damage degrees of the nutrient foramina in the femoral neck when the “In–Out–In” posterosuperior screw was placed in the different positions of the posterosuperior regions. We hypothesized that there was a risk zone of damaging nutrient foramina for the “In–Out–In” posterosuperior screws.

## Methods

### Subjects

We obtained 108 unpaired dry human cadaveric proximal femurs from the Department of Anatomy. General information regarding these dry specimens of adult femurs, such as age and gender, was unknown. Using a 3D scanner (Shanghai Digital Manufacturing Corp., Ltd., Shanghai, China), the surfaces of the proximal femur were scanned. An image resolution of 1,310,000 pixels was obtained by a scanning distance of 200 mm, precision of 0.01 mm, and measuring point of 0.04 mm. The digital 3D model was recorded from the proximal femur surface. Data were then processed using 3-matic 12.0 (Materialise) software.

### Identification and mark of nutrient foramina in femoral neck

**Fig. 1:**
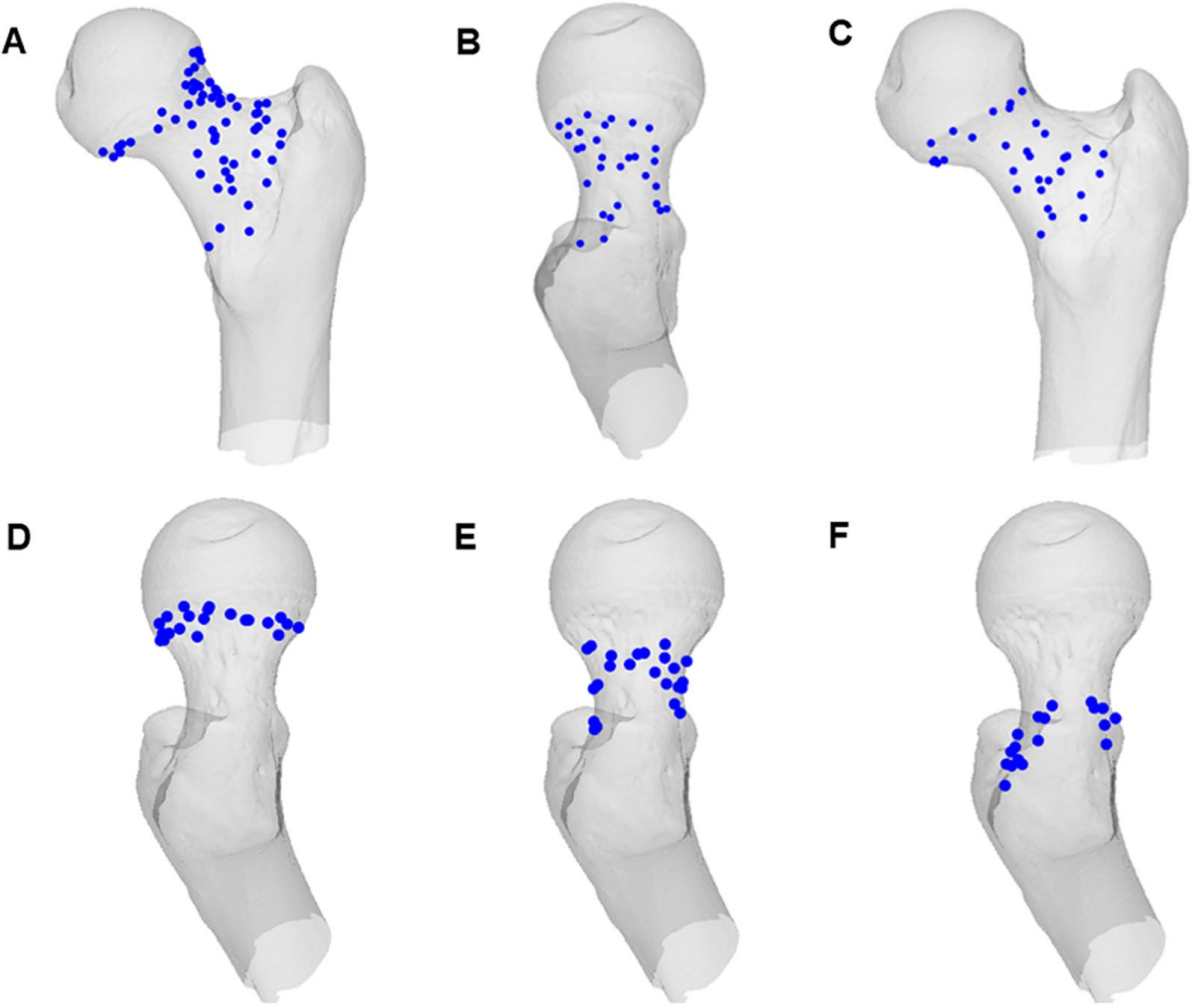
Nutrient foramina of the femoral neck in each 3D model. All nutrient foramina were identified and marked (**A**). Nutrient foramina were labeled based on their corresponding positions, including the posterior–superior (**B**) and non-posterior–superior region (**C**) regions. Each region was divided into subcapital (**D**), transcervical (**E**), and basicervical (**F**)

Based on the center of the nutrient foramen, the positions of nutrient foramen were defined in 3D models [10]. The points were used to mark the positions of nutrient foramen in the proximal femur surface. Identifications and marks were made for each of the nutrient foramina in the femoral neck (Fig. 1A). The femoral neck was divided into two main regions, including the posterior–superior region (Fig. 1B), covered by the upper (lateral) retinacula, and the non-posterior–superior region (Fig. 1C), covered by the anterior and lower (medial) retinacula [11]. Each region was divided into three parts, which were subcapital (Fig. 1D), transcervical (Fig. 1E), and basicervical (Fig. 1F). The label of nutrient foramen was performed according to the corresponding location.

### Radiograph simulation [1]

There were four synchronized windows available in the 3-matic software. One window was set as the front view, while the other was set as the lateral view. When the image opacity was set to 50%, the front and the lateral views, respectively, appeared as an anteroposterior (AP) and a lateral radiograph of the femoral neck.

**Fig. 2:**
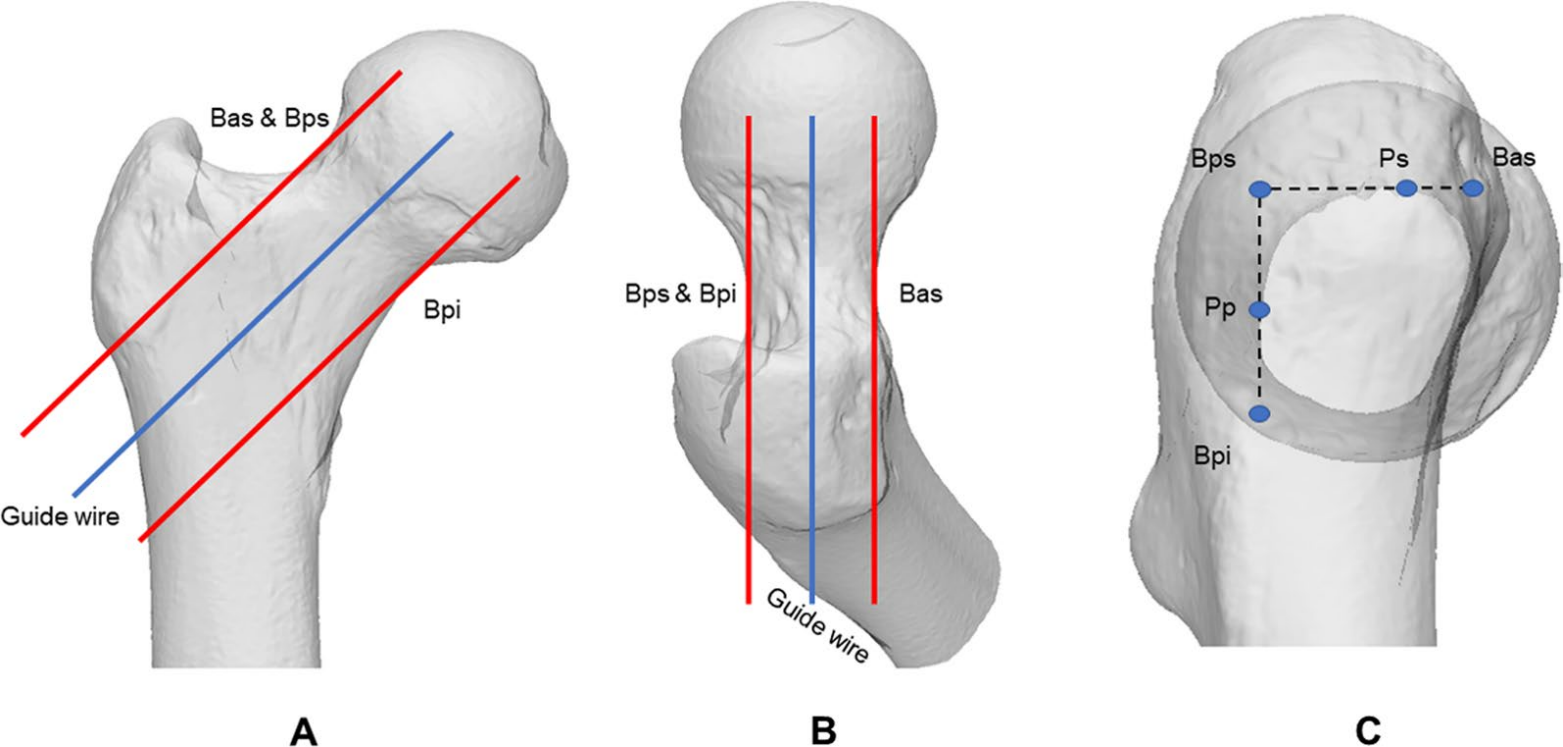
Simulated radiographs. In the AP view, Bas and Bps were tangential to the superior curve of the femoral neck and Bpi was tangential to the inferior curve (**A**). In the lateral view, Bas was tangential to the anterior curve of the femoral neck and Bps and Bpi were coincidental and tangential to the posterior curve (**B**). The axial view showed the site of Bas, Bps, Bpi, Ps, and Pp (**C**)

Based on two points in three-dimensional space, the femoral neck axis was determined in the AP view. One point was at the center of the femoral neck's smallest cross section, and the other was at the center of the femoral head. To correct the femoral neck anteversion angle after the axis was created, the femoral neck was rotated to ensure that the above two points were on the axis in the lateral view. The screw placement based on the guide wire was parallel to the femoral neck axis (Fig. 2).

Three red lines were constructed to set auxiliary lines for marking the femoral neck. The boundary posterior–superior (Bps) line was parallel to the femoral neck axis in AP and lateral radiographs, and the Bps was also tangential to the posterior and superior curves of the femoral neck in the lateral and AP radiograph, respectively. The boundary posterior–inferior (Bpi) and anterior–superior (Bas) lines were set in a similar principle. Finally, the simulated view was rotated until the boundary lines were observed as dots in order to create a projective graph along the femoral neck axis. So, the point of the Bps (P_Bps_) and two intersection points (the superior point, Ps; the posterior point, Pp) between the femoral neck curve and two lines, between three dots, were determined (Fig. 2).

### Coordinate extraction

**Fig. 3:**
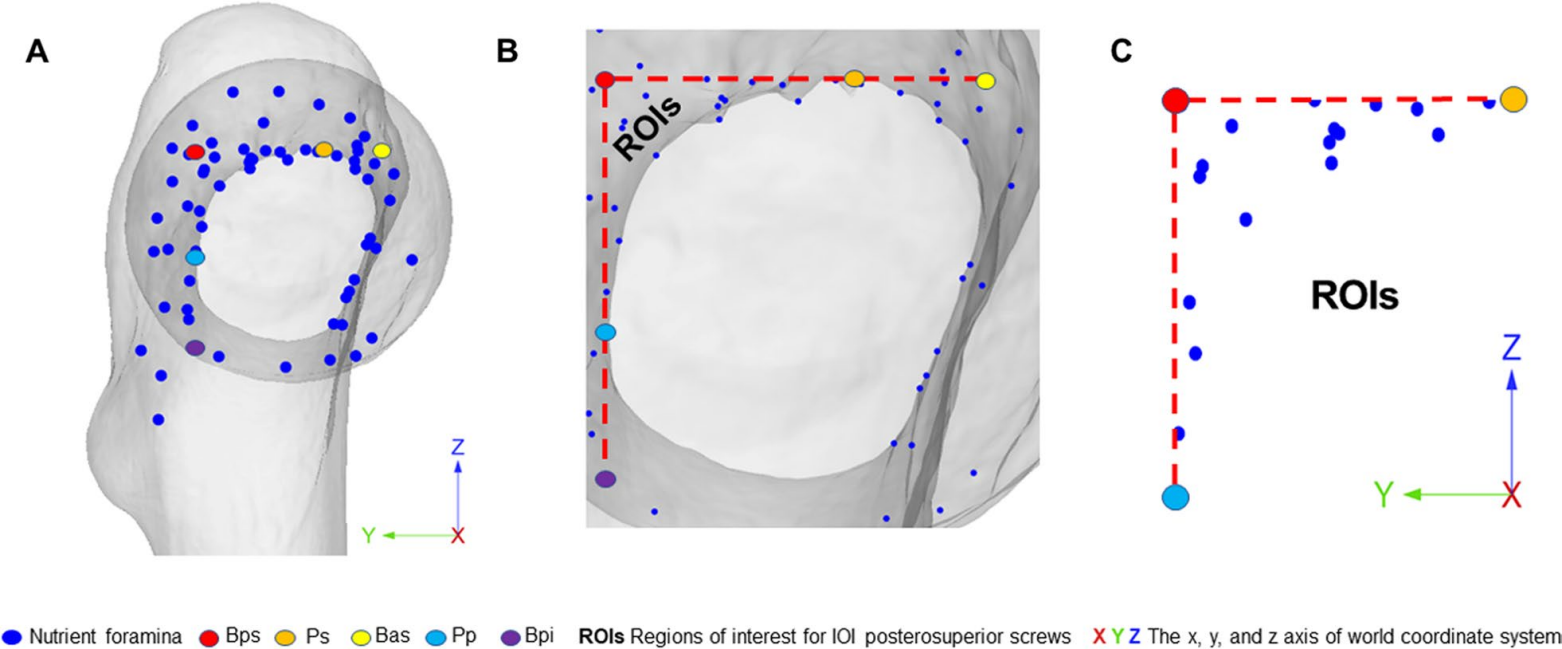
Digital simulation of nutrient foramina and marker points. In the world coordinate system, the femoral neck axis was aligned parallel to the *x*-axis, and all points were present as the values of *Y* and *Z* coordinates (*y*, *z*) (**A**). The ROIs were determined by three points (P_Bps_, Ps, and Pp) (**B**). The boundary of ROIs was determined by identifying the site of P_Bps_, Ps, and Pp (**C**)

The femoral neck axis was aligned parallel to the *x*-axis in the world coordinate system of 3-matic software. Also, all points in the femoral neck and boundary lines as a whole moved with the femoral neck axis. Then, the *Y* and *Z* coordinates of all points were extracted as (*y*, *z*) (Fig. 3A). For analysis, the data were imported into Microsoft Excel.

### Regions of interest (ROIs) for “In–Out–In” posterosuperior screws

The “In–Out–In” posterosuperior screws can damage nutrient foramina in the posterior and superior regions of the femoral neck. These regions were defined as regions of interest (ROIs) for “In–Out–In” posterosuperior screws. In the projective graph, the ROIs were determined by three points (P_Bps_, Ps, and Pp) (Fig. 3B). Thus, the y coordinate of points of nutrient foramina in ROIs was ranging from that of P_Bps_ to Ps, and the z coordinate of points of nutrient foramina in ROIs was ranging from that of Pp to P_Bps_ (Fig. 3C).

### Damage degrees analysis of nutrient foramina in different screw placements

**Fig. 4:**
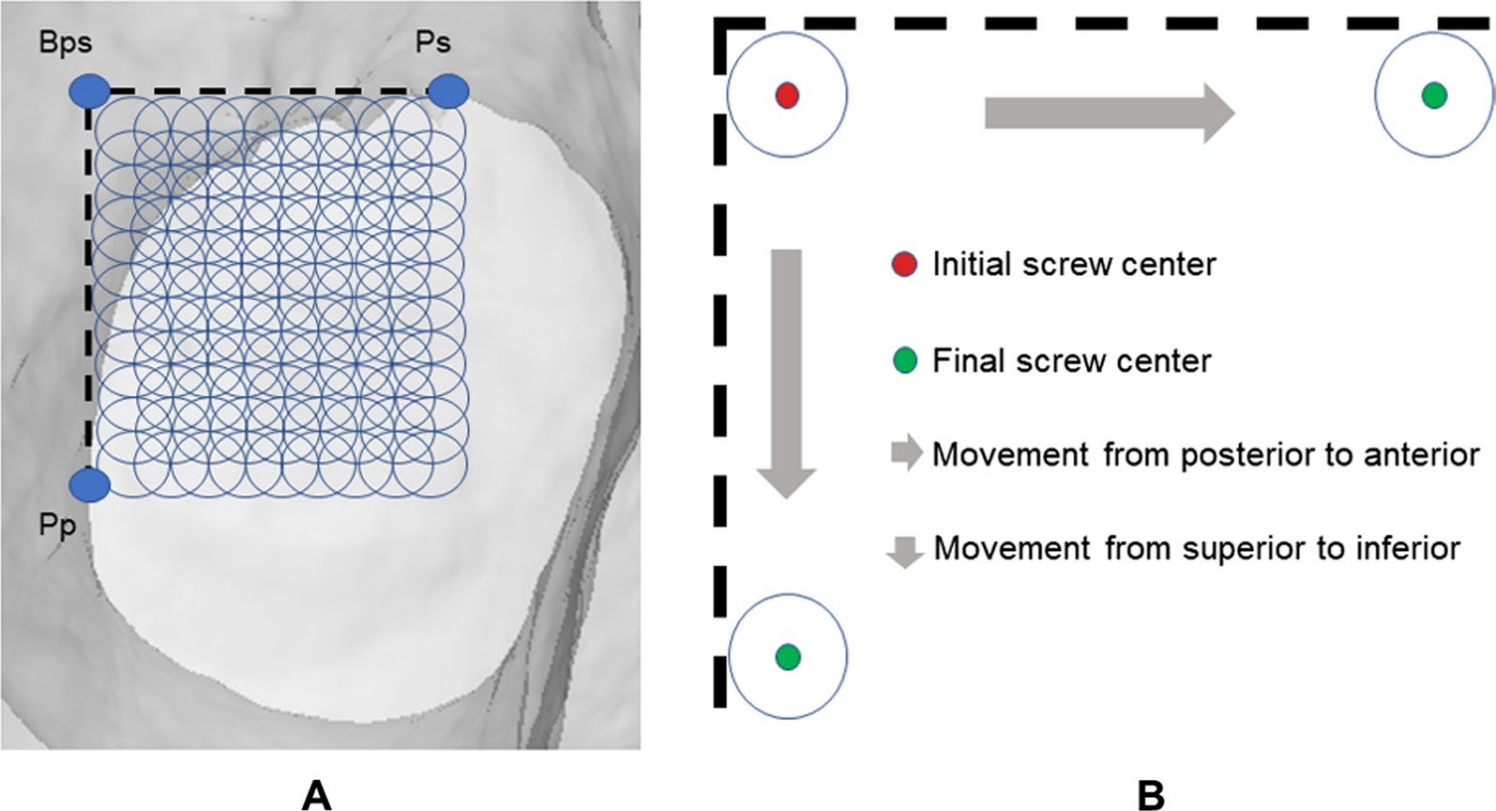
Schematic diagram of different placement positions of IOI posterosuperior screws with 6.5 mm diameter. The start and end positions of the screw were shown, including PBps, Ps, and Pp, and the screw was simulated moved at the travel distance of a radius of the screw, 3.25 mm diameter (**A**). The direction of the movement was in order, firstly horizontal and then vertical (**B**)

Nutrient foramina damaged by 6.5-mm-diameter screws were sought. The standardized coordinate of the screw center position was (*y*, *z*). The start and end positions of the “In–Out–In” posterosuperior screw were defined as the positions intersecting the critical points of ROIs, including P_Bps_, Ps, and Pp. The travel distance of each screw movement was set to a radius of the screw, 3.25 mm (Fig. 4A). The direction of the movement was from posterior to anterior and then from superior to inferior (Fig. 4B). If the distance between nutrient foramina (*y*_nf_, *z*_nf_) and the center of the screw was less than the radius of the screw, the nutrient foramina were damaged. The initial position of the center of the screw was located in the anterior–inferior site of the P_Bps_ (*y*_Bps_, *z*_Bps_), and it was 3.25 mm far away from the P_Bps_ (Fig. 4B). Hence, the initial coordinate of the screw center was (*y*_Bps_ − 3.25, *z*_Bps_ − 3.25). The formula was as follows:

$$(y_{{{\text{nf}}}} - y_{{{\text{Bps}}}} - 3.25)^{2} + (z_{{{\text{nf}}}} - z_{{{\text{Bps}}}} - 3.25)^{2} \le 3.25^{2}$$

### Statistical analysis

A preliminary analysis was conducted using Microsoft Excel at the descriptive level. The number of nutrient foramina was counted based on that of corresponding coordinates. The number of damaged nutrient foramina in ROIs was estimated according to the above formula when the “In–Out–In” posterosuperior screw was placed in a different position. The data in each region were expressed as percentages of nutrient foramina per ROI or femoral neck. Values presented were mean ± standard deviation. Secondly, the data analysis was then performed with SPSS software (IBM SPSS Statistics, version 26, IBM Corp.). The statistical significance of paired data was tested using paired *t*-tests. *P* < 0.01 was considered to be statistically significant.

## Results

In the femoral neck, the distribution ranking of nutrient foramina was in the subcapital, the transcervical, and the basicervical, with a mean percentage of the local number over the total number of 35.56%, 34.94%, and 29.51%, respectively. In the subcapital region, the mean percentage was 15.75% in the superior–posterior area and 19.81% in the non-superior–posterior area. In the transcervical region, a mean of 15.33% of nutrient foramina was found in the superior–posterior area and 19.61% in the non-superior–posterior area. In the basicervical region, the 12.00% of the nutrient foramina were in the superior–posterior area and the 17.51% were in the non-superior–posterior area.

In the ROIs, most nutrient foramina were located in the transcervical region and the least in the subcapital region (Table 1). In the subcapital, transcervical, and basicervical regions, the mean percentage of the number of the nutrient foramina over the total number in the normal femoral neck was 3.41%, 13.15%, and 6.88%, respectively, and the mean percentage of the number of the nutrient foramina over the total number in the ROIs was 13.99%, 56.36%, and 29.65%, respectively. In addition, most nutrient foramina were located in the superior–posterior area of the femoral neck (Table 1).

**Table 1:** The distribution of nutrient foramina of the “In–Out–In” region in the femoral neck

Region	ROI	
	Total^a^ (%)	Local^b^ (%)
*Subcapital*		
Superior–posterior	3.34 ± 2.41	13.69 ± 8.34
Non-superior–posterior	0.07 ± 0.37	0.30 ± 1.81
*Transcervical*		
Superior–posterior	10.14 ± 3.32	44.08 ± 12.26
Non-superior–posterior	3.01 ± 2.23	12.28 ± 8.43
*Basicervical*		
Superior–posterior	6.17 ± 2.11	26.83 ± 8.31
Non-superior–posterior	0.71 ± 1.09	2.82 ± 4.33

*ROI* The region of interest of the “In–Out–In” region

^a^The percentage of the number of the nutrient foramina over the total number in the femoral neck

^b^The percentage of the number of the nutrient foramina in the “In–Out–In” region over the total number in the femoral neck

There were six placements of “In–Out–In” posterosuperior screws that can damage nutrient foramina. Their movement directions and distances from the initial screw center along the z-axis and the y-axis were as follows: (0, 0), [(anterior, 3.25 mm), 0], [0, (inferior, 3.25 mm)], [(anterior, 3.25 mm), (inferior, 3.25 mm)],

[0, (inferior, 6.5 mm)], and [0, (inferior, 9.75 mm)]. In the location of [0, (inferior, 6.5 mm)], nutrient foramina of only three of 108 subjects were damaged, with the number ranging from one to three. In the location of [0, (inferior, 9.75 mm)], one nutrient foramina of only one of 108 subjects were damaged. Thus, these last two conditions were the most extremely rare, and the former four conditions were used for the subsequent analysis.

**Fig. 5:**
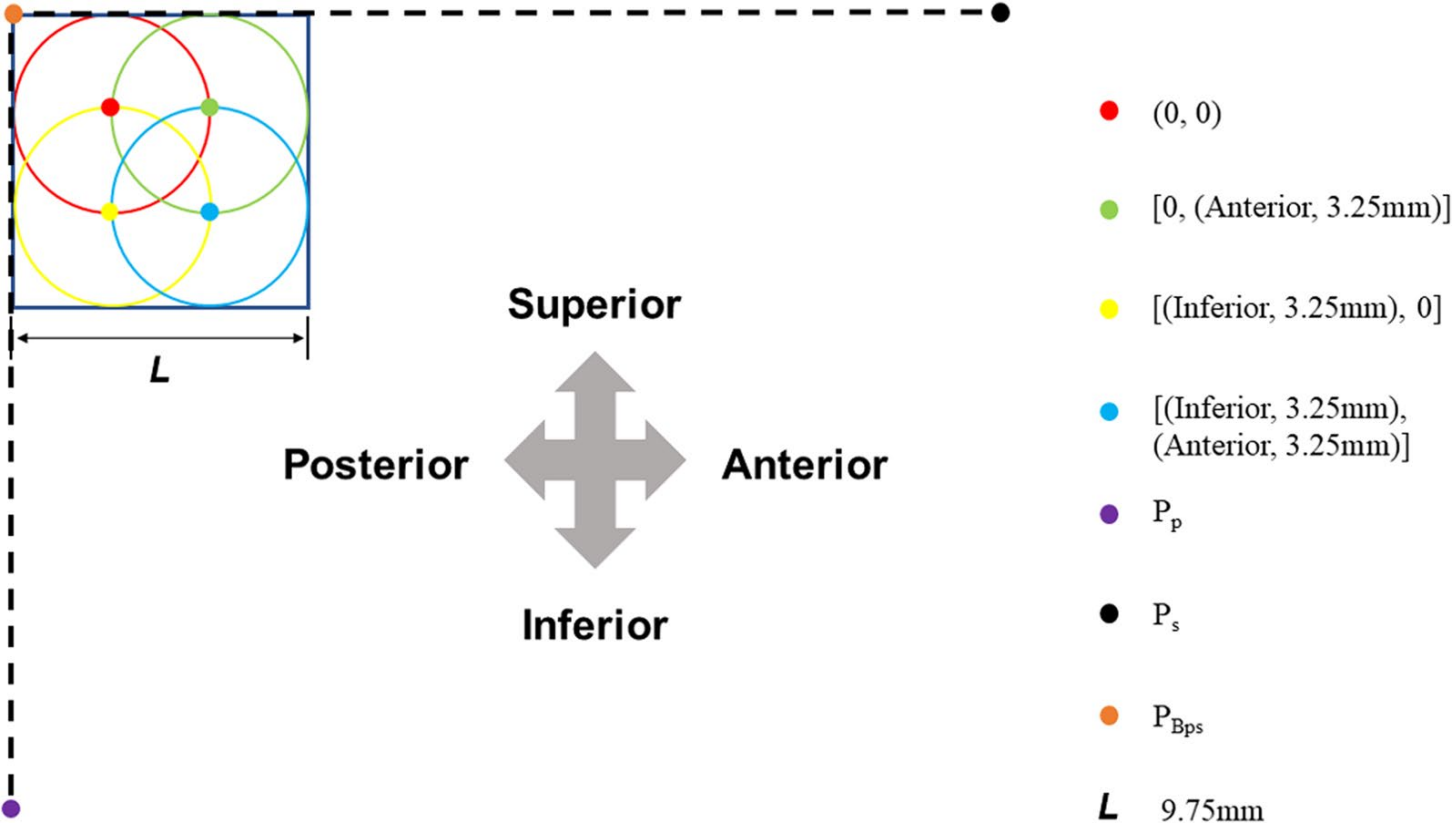
A schematic representation of the risk zone for IOI posterosuperior screws in the posterosuperior femoral neck

The results showed that the extent of nutrient foramina decreased in different locations in the following order: (0, 0) > [0, (inferior, 3.25 mm)] > [(anterior, 3.25 mm), 0] > [(anterior, 3.25 mm), (inferior, 3.25 mm)] (Table 2). When the screw was placed in the location of (0, 0) and [(anterior, 3.25 mm), 0], the decrease in the nutrient foramina was statistically significant in the transcervical area and superior–posterior area of the subcapital and basicervical regions (*P* < 0.0001). In the condition of [0, (inferior, 3.25 mm)], the decrease in the nutrient foramina was statistically significant in the superior–posterior area of the femoral neck (*P* < 0.0001). In the condition of [(anterior, 3.25 mm), (inferior, 3.25 mm)], the nutrient foramina reduction was statistically significant in the superior–posterior area of subcapital, transcervical, and basicervical regions, with a *P*-value of < 0.0001, 0.0026, and < 0.0001, respectively. Therefore, the “In–Out–In” posterosuperior screw mostly damaged the nutrient foramina in the superior–posterior regions. When the screw center was in the surrounding site of P_Bps_, a risk zone, there was a risk of damaging nutrient foramina. This zone was located in a square of ROIs with an edge length of 9.75 mm, and P_Bps_ was its posterosuperior vertex (Fig. 5).

**Table 2:** Comparison of the percentage of the nutrient foramina between the normal and In–Out–In groups

Region	Normal	ROI							
		Anterior 0 Inferior 0		Anterior 3.25 Inferior 0		Anterior 0 Inferior 3.25		Anterior 3.25 Inferior 3.25	
	Total^a^ (%)	Total^a^ (%)	*P*-value^b^	Total^a^ (%)	*P*-value^b^	Total^a^ (%)	*P*-value^b^	Total^a^ (%)	*P*-value^b^
*Subcapital*									
Superior–posterior	15.75 ± 3.07	13.43 ± 3.23	< 0.0001	14.41 ± 3.07	< 0.0001	13.77 ± 3.13	< 0.0001	14.72 ± 3.05	< 0.0001
Non-superior–posterior	19.81 ± 3.88	19.76 ± 3.88	0.1699	19.75 ± 3.86	0.1002	19.81 ± 3.88	–	19.79 ± 3.89	0.3196
*Transcervical*									
Superior–posterior	15.33 ± 3.83	12.07 ± 3.44	< 0.0001	14.58 ± 3.82	< 0.0001	14.52 ± 3.61	< 0.0001	15.18 ± 3.79	0.0026
Non–superior–posterior	19.61 ± 4.07	19.19 ± 4.06	< 0.0001	19.22 ± 4.05	< 0.0001	19.57 ± 4.08	0.3196	19.59 ± 4.07	0.3196
*Basicervical*									
Superior–posterior	12.00 ± 2.83	9.50 ± 3.05	< 0.0001	11.24 ± 2.74	< 0.0001	11.14 ± 2.74	< 0.0001	11.73 ± 2.78	< 0.0001
Non-superior–posterior	17.51 ± 3.31	17.46 ± 3.35	0.0878	17.49 ± 3.34	0.3196	17.51 ± 3.31	–	17.51 ± 3.31	–

*ROI* The region of interest of the “In–Out–In” region

^a^The percentage of the number of the nutrient foramina over the total number in the normal femoral neck

^b^Paired *t*-test was used to compare data before and after the injury of nutrient foramina in “In–Out–In” groups

## Discussion

This study demonstrated that there was a risk zone of damaging nutrient foramina when the posterosuperior screws were “In–Out–In” in the management of femoral neck fractures. And, this zone was located around the site of P_Bps_ based on traditional AP and lateral radiographs. The impacts of the “In–Out–In” posterosuperior screws, in different placements, on nutrient foramina were illustrated in this study, and our results confirmed that not all “In–Out–In” posterosuperior screws can damage the blood supply of the femoral head. Therefore, the risk zone in the posterosuperior femoral neck should be avoided when intraoperatively placing the posterosuperior screw for femoral neck fracture fixation.

Most nutrient foramina of ROIs were located in the transcervical region, and the damage of nutrient foramina from “In–Out–In” posterosuperior screws was likely to be mild. Consistent with previous reports, most nutrient foramina were located in the subcapital region of the femoral neck [10, 12]. In addition, nutrient foraminal density was significantly higher in the posterior–superior region, mainly covered by the superior retinacular arteries [10–12]. When the superior retinacular artery was damaged, there was an increased risk of femoral head avascular necrosis [13]. Due to the elasticity of the blood vessel and its surrounding tissues, it is difficult to judge whether the blood vessels are damaged by the “In–Out–In” posterosuperior screws. However, nutrient foramina were damaged when the screw was placed in their corresponding positions. Interestingly, the least percentage, 3.34% of the total and 13.69% of the local, of nutrient foramina was observed in the subcapital area of the posterior–superior region in ROIs (Table 1). Therefore, ROIs may not be a “minefield” of “In–Out–In” posterosuperior screws.

The risk zone in this study could provide a reference for ideal screw positions in the clinical treatment of femoral neck fractures. With the insufficient precision of CT or MRI, the nutrient foramina could not be directly assessed in the clinic. In this study, there were four main locations of “In–Out–In” posterosuperior screws, which caused a significant reduction in nutrient foramina. The distribution of the terminal branch of the nutrient vessels matched that of the nutrient foramina [10]. Therefore, when the screws were away from these dangerous locations, the risk of damaging the blood supply could be decreased. A risk zone, determined by these locations, can provide surgeons with more placement options for the posterosuperior screw for patients with femoral neck fractures.

Our findings could provide a theoretical basis for updating the treatment principle, while the unacceptable concept of all “In–Out–In” was still used in clinical practice and research. Previous studies reported a variety of attempts to avoid the presence of the “In–Out–In” status of the posterosuperior screw, but there was still a lack of consensus on preventive measures [4–8]. Kumar et al. introduced an optimal radiographic projection to exclude “In–Out–In” screws in the femoral neck by aligning the C-arm close to the angles 55° and 124° for anterosuperior and posterosuperior surfaces, respectively [4]. Likewise, Aibinder et al. demonstrated that the sequential fluoroscopic rollover images were highly sensitive to detecting an “In–Out–In” position after placing the posterosuperior guide pin into the femoral neck, with an optimal rollover degree of 40° [5]. Terhune et al. found that a − 15° rollunder fluoroscopic view can be added to improve the identification of “In–Out–In” screws [6]. However, these promising preliminary findings could be better validated by further cadaveric or clinical studies before wide clinical application. Recently, the piriformis fossa landmark on the AP view was considered a reference site for avoiding the presence of an “In–Out–In” posterosuperior screw [7, 8]. Also, there were notable challenges to the clear radiographic visualization of the piriformis fossa by the intraoperative C-arm X-ray machine. Therefore, conventional radiograph views cannot be completely replaced to date, and it was still the surgeons’ first choice due to its convenience and efficiency in intraoperative fluoroscopic visualization. In addition, direct screw placement in ROIs could be another potential therapeutic strategy for femoral neck fractures.

The “In–Out–In” posterosuperior screws were traditionally inevitable, but this screw may result in better biomechanical properties. Due to the complex osseous anatomy of the femoral neck, a high incidence of “In–Out–In” posterosuperior screws was observed based on conventional AP and lateral radiograph views [2, 3]. Most “In–Out–In” posterosuperior screws can be confirmed by anatomical inspection in cadavers study or clinical CT scans, although radiographically desired. However, potential gains from these screws can be expected for the fracture fixation of the femoral neck [9]. “In–Out–In” posterosuperior screws provided multi-cortical rigid fixation with four-point stability if partially extraosseous, and three screws with the “In–Out–In” posterosuperior one formed a larger cross-sectional area, compared to all screws located within the femoral neck fracture cortex [9].

This study has several limitations. Firstly, intact dry human proximal femurs were used to generate the digital data. Therefore, before considering the posterosuperior screw placement, the anatomic reduction is essential in a clinical situation. Secondly, the 6.5-mm-diameter screw was only chosen for analysis in this study, although it was the most commonly used one. When considering other screws with a greater diameter in some special cases, screw locations that were away from the risk zone could be recommended.

## Conclusion

Not all “In–Out–In” posterosuperior screws can damage the nutrient foramina of the femoral neck. To minimize iatrogenic damage to the blood supply of the femoral head, screw positions could be assessed in AP and lateral radiographs using a risk zone. With a biomechanical advantage to the fracture site, the “In–Out–In” posterosuperior screw can be applied to fix femoral neck fracture when feasible in clinical practice. Therefore, this study provided surgeons with more alternatives for screw placement in the posterosuperior femoral neck.

## Data Availability

The datasets used and/or analyzed during the current study are available from the corresponding author on reasonable request.

## Abbreviations

IOI: In−Out−In
ROI: Region of interest
AP: Anteroposterior
Bps: Boundary posterior–superior
Bpi: Boundary posterior–inferior
Bas: Boundary anterior–superior

## References

[1] Zhang YQ, Chang SM, Huang YG, Wang X (2015). The femoral neck safe zone: a radiographic simulation study to prevent cortical perforation with multiple screw insertion. J Orthop Trauma.

[2] Hoffmann JC, Kellam J, Kumaravel M, Clark K, Routt MLC, Gary JL (2019). Is the cranial and posterior screw of the “inverted triangle” configuration for femoral neck fractures safe?. J Orthop Trauma.

[3] Yuan BJ, Shamaa MT, Aibinder WR, Parry JA, Cross WW, Barlow JD (2020). High incidence of “in–out–in” posterosuperior screws after cannulated screw fixation of femoral neck fractures. Eur J Orthop Surg Traumatol.

[4] Kumar A, Jameel J, Qureshi OA, Kumar M, Haider Y, Das S (2021). Modified radiographic views to prevent the anterosuperior and posterosuperior bony violation during screw fixation of femoral neck fractures. Eur J Orthop Surg Traumatol.

[5] Aibinder WR, Yuan BJ, Cross WW, Parry JA (2020). Sequential fluoroscopic rollover images reliably identify “in–out–in” posterosuperior screws during percutaneous fixation of femoral neck fractures. Eur J Orthop Surg Traumatol.

[6] Terhune EB, Polce EM, Williams JC (2022). A novel fluoroscopic view for improved assessment of the safety of the posterosuperior screw in femoral neck fracture fixation. J Bone Jt Surg Am.

[7] Kuttner NP, Hoggard TM, Cancio-Bello AM, Hidden KA, Yuan BJ, Adams JD (2023). The use of the piriformis fossa radiographic Landmark to predict “In–Out–In” placement of the posterosuperior femoral neck screw. J Orthop Trauma.

[8] Adams JDJ, Walker JB, Loeffler M (2022). Avoid the In–Out–In posterosuperior femoral neck screw: the use of the piriformis fossa radiographic landmark. J Orthop Trauma.

[9] Zhang J, Lou Z, Tang X (2023). Biomechanical assessment of the posterosuperior cannulated screw in–out–in in femoral neck fracture. Asian J Surg.

[10] Wu S, Quan K, Wang W, Zhang Y, Mei J (2022). 3D mapping of bone channel of blood supply to femoral head in proximal femur. Front Surg.

[11] Mei J, Ni M, Wang G, Jia G, Liu S, Cui X (2017). Number and distribution of nutrient foramina within the femoral neck and their relationship to the retinacula of Weitbrecht: an anatomical study. Anat Sci Int.

[12] Kamath V, Gupta C (2022). Morphological study on distribution of nutrient foramina in femoral neck in relation to retinacula of weitbrecht with its surgical implications. J Orthop.

[13] Lazaro LE, Klinger CE, Sculco PK, Helfet DL, Lorich DG (2015). The terminal branches of the medial femoral circumflex artery: the arterial supply of the femoral head. Bone Jt J.

